# Glucagon-like peptide-1 receptor agonists (GLP-1RAs) as treatment for nicotine cessation in psychiatric populations: a systematic review

**DOI:** 10.1186/s12991-024-00527-9

**Published:** 2024-11-11

**Authors:** Serene Lee, Maggie Li, Gia Han Le, Kayla M. Teopiz, Maj Vinberg, Roger Ho, Hezekiah C. T. Au, Sabrina Wong, Kyle Valentino, Angela T. H. Kwan, Joshua D. Rosenblat, Roger S. McIntyre

**Affiliations:** 1 Brain and Cognition Discovery Foundation, 77 Bloor Street West, Suite 617, Toronto, ON M5S 1M2 Canada; 2 Mood Disorder Psychopharmacology Unit, University Health Network, Toronto, Canada; 3 Institute of Medical Science, University of Toronto, Toronto, Canada; 4 Department of Pharmacology and Toxicology, University of Toronto, Toronto, Canada; 5 Department of Health Sciences, Queen’s University, Kingston, Canada; 6 Faculty of Medicine, University of Ottawa, Ottawa, ON Canada; 7 Department of Psychological Medicine, Yong Loo Lin School of Medicine, Singapore, Singapore; 8 Institute for Health Innovation and Technology (iHealthtech), National University of Singapore, Singapore, Singapore; 9 Division of Life Science (LIFS), Hong Kong University of Science and Technology (HKUST), Clear Water Bay, Hong Kong, Hong Kong; 10 The Early Multimodular Prevention and Intervention Research Institution (EMPIRI), Mental Health Centre, Northern Zealand, Copenhagen University Hospital-Mental Health Services CPH, Copenhagen, Denmark; 11 Department of Clinical Medicine, Faculty of Health and Medical Sciences, University of Copenhagen, Copenhagen, Denmark; 12 Department of Psychiatry, University of Toronto, Toronto, Canada

**Keywords:** GLP-1, Nicotine use disorder, Exenatide, Liraglutide, Nicotine, Semaglutide, Dulaglutide, Lixisenatide, Tirzepatide

## Abstract

**Background:**

Nicotine use and nicotine use disorder (NUD) are the leading causes of preventable death in the United States. Persons with mental disorders  (e.g., bipolar disorder) are differentially susceptible to nicotine use. Glucagon-like peptide-1 receptor agonists (GLP-1RAs) are indicated for type 2 diabetes mellitus (T2DM) and obesity and show preliminary evidence of efficacy in addiction-related behaviours. Herein, we synthesize extant preclinical and clinical evidence evaluating the effect of GLP-1RAs on neurobiological systems and behaviours salient to nicotine consumption and cessation.

**Methods:**

Online databases (MedLine, Embase, AMED, PsychINFO, JBI EBP Database, PubMed, Web of Science, Google Scholar) were searched from inception to May 21, 2024. Relevant studies were also extracted from the reference lists of the obtained articles. All articles were screened against inclusion and exclusion criteria.

**Results:**

Administration of GLP-1RAs reduced nicotine self-administration and nicotine-seeking behaviour in animal models that, in some cases, is sustained beyond exposure to the agent. GLP-1RAs also mitigated post-nicotine cessation weight gain, craving, withdrawal, and hyperphagia. The preceding effects are attributable to modulation of reward-related brain regions (e.g., mesolimbic dopamine system), resulting in nicotine aversion. GLP-1RAs were also efficacious as adjunctive therapies [e.g., in combination with nicotine replacement therapies (NRTs)].

**Conclusion:**

The multi-effect characteristics in NUD paradigms provide a compelling rationale for large, adequately powered, long-term, randomized controlled trials of GLP-1RAs in the treatment and prevention of NUD. The replicated effect on mitigating post-nicotine cessation weight gain is a differentiating feature of GLP-1RAs from extant proven therapies for NUD.

## Background

Nicotine use and nicotine use disorder (NUD) are highly associated with excess and premature disease and mortality (e.g., cardiovascular disease), which differentially affect persons with mental disorders (e.g., bipolar disorder, schizophrenia)  [1–4]. It is further reported that substance use in persons with mental disorders is associated with nonadherence and less favourable treatment outcomes [5]. NUD is characterized by dependence, craving, withdrawal, and tolerance [3]. The Food and Drug Administration (FDA) has approved bupropion, a norepinephrine dopamine reuptake inhibitor (NDRI), varenicline, an α_4_β_2_ nicotinic agonist, as well as nicotine replacement therapies (NRTs) for NUD [6–9]. Notwithstanding, long-term smoking cessation rates continue to be suboptimal, and there is a need for alternative agents with improved profiles of long-term efficacy, acceptability, tolerability, and safety [6–9]. In addition, none of the existing therapeutics for NUD meaningfully mitigate post-nicotine cessation weight gain or psychotropic-drug related weight gain, which are common phenomena predisposing non-initiation of cessation efforts as well as return-to-smoking behaviour in both the general and psychiatric population [9].

Glucagon-like peptide-1 (GLP-1) is an endogenous neurotransmitter that plays a role in glucose homeostasis through insulin secretion and enhancing insulin sensitivity [10]. GLP-1 receptors are identified on pancreatic β-cells, but are also widely distributed throughout the central nervous system, such as the nucleus tractus solitarius (NTS) [11]. Exogenous GLP-1 receptor agonists (GLP-1RAs) mimic the effects of endogenous GLP-1. GLP-1RAs affect disparate physiological processes, including satiety and metabolism, with proven efficacy in promoting weight loss by affecting reward salience [11–13].

The effect that GLP-1RAs have on reward salience includes effects on neurotransmitter systems and regions that subserve reward through neurotransmitter release (e.g., ventral tegmental area, nucleus accumbens) [11]. In addition, GLP-1RAs affect dopamine's synaptic availability and dopamine reuptake transporter (DAT) expression, which is known to mediate motivation and reward behaviours [14].

Herein, we evaluate extant literature on the effects of GLP-1RAs on NUD and nicotine use by providing a comprehensive overview of downstream and neurobiological mechanisms involved in reward pathways. The overarching objective of this analysis, which derives from a working hypothesis of GLP-1RAs and their effect on NUD and nicotine administration is that GLP-1RAs represent a promising therapeutic for nicotine administration and additionally prevents and treats associated complications of nicotine discontinuation (e.g., hyperphagia, weight gain, metabolic disruption).

## Methods

### Search strategy

Following the 2020 Preferred Reporting Items for Systematic Reviews and Meta-Analyses (PRISMA) guidelines, a comprehensive search was conducted on MedLine, Embase, AMED, PsychINFO, JBI EBP Database, PubMed, Web of Science, and  Google Scholar from inception until May 21, 2024 [15]. The search employed the following terms: ("GLP-1" or "Glucagon-Like Peptide-1" or "GLP-1 Agonist" or "Glucagon-Like Peptide-1 Agonist" or "Semaglutide" or "Ozempic" or "Rybelsus" or "Wegovy" or "Dulaglutide" or "Trulicity" or "Exenatide" or "Byetta" or "Bydureon" or "Liraglutide" or "Lixisenatide" or "Tirzepatide" or "Mounjaro" or "Zepbound" or "Bydureon BCise" or "Adlyxin" or "Victoza" or "Saxenda") AND ("Nicotine"). Additional queries were performed on Google Scholar and through reference list checking to ensure the search was fully comprehensive.

### Eligibility and inclusion criteria

Relevant studies retrieved from the comprehensive search were screened based on the inclusion and exclusion criteria (Table 1). Using the Covidence platform, three reviewers (SL, ML, and GHL) independently screened studies first by title and abstract [16]. Articles found relevant by at least one reviewer subsequently underwent full-text screening, and any discrepancies were resolved through discussion.

**Table 1:** Eligibility criteria

Inclusion criteria	1. A primary or post-hoc analysis 2. Animal models (e.g., rats and mice), sample population consisting of humans 3. Animals must be administered with nicotine and glucagon-like peptide 1 (GLP-1) agonists (e.g., liraglutide, exendin) 4. Investigate the effect of GLP-1 and GLP-1 agonists on nicotine craving, 5. English language 6. Full-text article available online
Exclusion criteria	1. Secondary articles (e.g., literature reviews, systematic reviews, meta-analyses, posters, abstracts, guidelines, protocols and theses) 2. Full-text is not available

### Data extraction

Three independent reviewers (SL, ML, and GHL) extracted data from the relevant studies using a piloted data extraction template. Any discrepancies encountered during the extraction process were resolved through discussion. The data that was to be extracted was predefined and included the following details: (1) author(s), (2) participants, (3) intervention, (4) duration, and (5) outcome of interest(s) (Table 4).

### Quality assessment

The quality assessment was conducted independently by three independent reviewers (SL, ML, and GHL). The assessment of the risk of bias for the included studies was conducted using the Quality Assessment of Controlled Intervention Studies, adapted from the National Institute of Health (NIH) guidelines [17]. Randomized controlled trials were assessed with the Cochrane Risk of Bias Tool for Randomized Studies (RoB2) [18]. The potential risk of bias in animal studies was evaluated using the SYRCLE Risk of Bias tool [19]. Refer to Tables 2 and 3 for risk of bias assessment.

**Table 2:** Risk of bias assessment for preclinical studies

Study	Item										Quality rating
	1	2	3	4	5	6	7	8	9	10	
Egecioglu et al. (2013)	U	Y	U	Y	U	Y	Y	Y	Y	Y	Low
Tuesta et al. (2017)	Y	Y	Y	Y	Y	Y	Y	Y	Y	Y	Low
Herman et al. (2023	Y	Y	Y	Y	U	U	U	Y	Y	Y	Low
Falk et al. (2023)	Y	Y	Y	Y	Y	Y	Y	Y	Y	Y	Low
Shankar et al. (2023)	Y	Y	Y	Y	U	Y	U	Y	Y	Y	Low

Y = yes, U = unclear, N = no

**Table 3:** Risk of bias assessment for clinical studies

Study	Item					Quality rating
	1	2	3	4	5	
Yammine et al. (2021)	N	N	N	N	N	Low
Lengsfeld et al. (2023)	N	N	N	N	N	Low
Luthi et al. (2024)	N	N	N	N	N	Low

## Results

### Search results

**Fig. 1:**
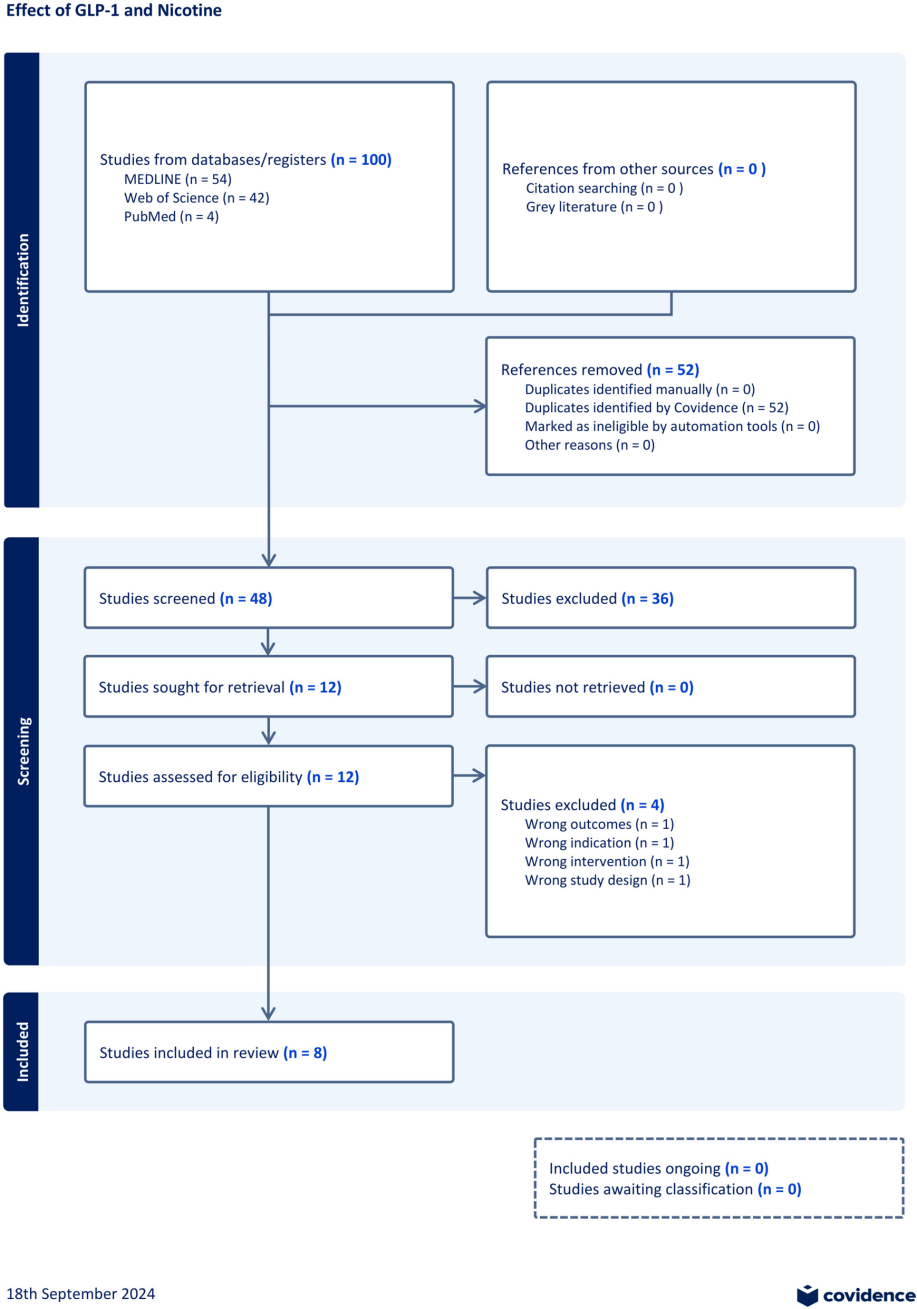
PRISMA flow diagram

From the aforementioned databases, 100 studies were retrieved from the search. After the removal of 52 duplicates, 48 studies underwent screening of their titles and abstracts. A total of 12 studies underwent full-text review and were screened based on the eligibility criteria. Subsequently, four studies were removed due to wrong outcomes (n = 2) and wrong study interventions (n = 2) (Fig. 1). Eight studies were included in this review; their results and experimental design are presented in Table 4.

**Table 4:** Descriptive characteristics of included preclinical and clinical studies

Author(s)	Participants	Intervention	Duration	Outcome(s) of interest
*Preclinical studies*				
Egecioglu et al. (2013)	NMRI mice (n = 8) per treatment combination (vehicle-vehicle, Ex4-vehicle, vehicle- nicotine or Ex4-nicotine) administered intraperitoneal nicotine	Ex-4 (2.4 μg/kg) administered intraperitoneally 10 min before nicotine administration	8 days	Ex-4 administered into nucleus accumbens reduced reward induced by nicotine and food, and is important for attenuation of nicotine-induced activation of the mesolimbic dopamine system. Ex-4 was also reported to attenuate hyperphagia
Tuesta et al. (2017)	Various groups including wild-type and Glp1r knockout mice administered nicotine. Numbers for each specific group varied (n = 10–12)	Sitagliptin (10 mg/kg) or Ex-4 (≤ 10 µg/kg)	Not stated	GLP-1RAs decreased nicotine intake. The IPN was shown to be important in nicotine avoidance by the MHb-IPN circuit, being a substrate for the inhibitory actions of GLP-1 on nicotine uptake
Herman et al. (2023)	Male (n = 63) and female (n = 58) rats self-administered intravenous nicotine	Liraglutide (25 μg/kg, i.p.) daily	21 days	Nicotine-induced withdrawal, hyperphagia, and body weight gain was prevented/ minimized in these rats with no malaise-like effects
Falk et al. (2023)	Diet-induced male obese mice (n = 8) administered nicotine once daily	Liraglutide (10 nmol/kg) subcutaneously once daily	16 days	Co-administration of nicotine and liraglutide synergistically lowered body weight. Significant reductions in food intake was also observed. Nicotine-induced dopamine release was attenuated by liraglutide, suggesting alterations in reward processing pathways
Shankar et al. (2023)	Experiment 1: male (n = 10) and female (n = 10) rats Experiment 2: male (n = 6) and female (n = 6) rats Experiment 3: male (n = 6) and female (n = 6) rats. All rats were administered nicotine	7-36amide (20 μg/kg) subcutaneously daily	Experiment 1: 14 days Experiment 2: 5–7 days Experiment 3: 5–7 days	Nicotine injection decreased circulating levels of GLP-1 and the drug’s potential to mitigate obesity may have a role in regulating the increased food intake observed in nicotine-dependent rats
*Clinical studies*				
Yammine et al. (2021)	84 Prediabetic and/or overweight smokers were randomized to control (n = 42) or experimental (n = 42)	Extended-release exenatide (2 mg) subcutaneously once weekly adjunctively with a 21 mg nicotine patch and smoking cessation counseling	6 weeks	Participants receiving exenatide reported reduced cravings and withdrawal symptoms compared to the placebo group. Exenatide adjunct therapy showed potential in reducing post-cessation weight gain. The primary outcome was the rate of smoking abstinence verified by expired carbon monoxide (CO) levels
Lengsfeld et al. (2023)	255 Participants were randomized to control (n = 128) or experimental (n = 127)	Dulaglutide (1.5 mg) subcutaneously once weekly adjunctive to standard of care smoking cessation therapy	12 weeks	The biochemically confirmed 7-day point prevalence abstinence rate at 12 weeks was 27% in the dulaglutide group compared to 18% in the placebo group. The dulaglutide group also experienced less weight gain post-nicotine cessation as well as reduced cravings
Luthi et al. (2024)	255 participants were randomized to control (n = 128) or experimental (n = 127)	Dulaglutide (1.5 mg) subcutaneously once weekly adjunctive to standard of care smoking cessation therapy	12 months	The point-prevalence abstinence rate at 52 weeks was 32% in the dulaglutide group and in the placebo group, showing no significant difference. The placebo group had an average weight gain of + 3.1 kg, while the dulaglutide group had an average weight gain of + 2.8 kg. Both groups experienced a decrease in nicotine cravings from baseline to week 12

### Methodological quality

Herein, we also assessed animal studies to complement the clinical research findings. It was noted that some of these studies did not adequately address certain critical aspects, not all studies clearly indicated random selection methods, and some did not specify how animals were chosen for outcome assessment. Specifically, two studies were found to have gaps in addressing blinding of caregivers/investigators and random selection for outcome assessment. All publications exhibited a low risk of bias across various domains (Tables 2, 3).

The included clinical studies consistently reported whether blinding was maintained for researchers and participants and whether allocation to experimental groups was randomized. None of the included human studies was found to have a high risk of bias (Table 3).

### Preclinical data

A total of five preclinical studies were identified evaluating the effect of GLP-1RA monotherapy on animal models of NUD. Rodents were allowed self-administered nicotine, forming nicotine-seeking and withdrawal habits [20, 21]. Herman et al. and Egecioglu et al. reported that liraglutide and exendin-4, respectively, attenuated self-administration and reinstatement in these rodents, while repeated administration of GLP-1RAs reduced the occurrence of withdrawal-induced hyperphagia and post-smoking cessation weight gain [20, 21]. Herman et al. found that during subsequent reinstatement sessions, total lever responses releasing nicotine were significantly lower in rats treated with liraglutide compared to the vehicle-treated controls [F(1, 24) = 60.86, p < 0.0001] [20]. Egecioglu et al. further reported that exendin-4 attenuated nicotine-induced dopamine release [21].

Additionally, Egecioglu et al. examined the impact of exendin-4 on nicotine-induced conditioned place preference, a behavioural paradigm that proxies drug reward properties [21]. Mice showed a significant preference for the nicotine-paired compartment, which was reversed by exendin-4 administration (p ≤ 0.05) [21].

The capability of GLP-1RAs to cross the blood–brain barrier suggests that the physiological role of these agents may extend beyond that of glucose homeostasis and food intake as a potential pharmacotherapy for NUD [21]. A separate study using murine models reported that 79% of GLP-1 neurons in the NTS were activated by nicotine exposure, which further reduced nicotine consumption [22]. The aforementioned findings were reported to be subserved by the medial habenula-interpeduncular nucleus (MHb-IPN) [22]. The MHb-IPN circuit has downstream targets that promote nicotine aversion, prompted by GLP-1 release from NTS terminals that stimulate habenular terminals, although such targets are currently unknown [22]. Additionally, mice treated with exendin-4 under a fixed ratio schedule of reinforcement exhibited a consistent inhibitory effect on nicotine consumption compared to the control [22].

Similarly, Falk et al. demonstrated attenuation of nicotine-induced dopamine release by liraglutide administration  [23]. The combination of nicotine and liraglutide increased neuronal activity in several brain regions involved in body weight regulation [23]. The combination administration synergistically increased c-Fos expression in the ventral tegmental area and nucleus accumbens [23].

Among patients with a high susceptibility for post-nicotine cessation weight gain, as well as risk for psychotropic-drug related weight gain, GLP-1RAs represent a viable option [24]. We identified one behavioural animal study that evaluated the association between metabolic change and nicotine consumption. Shankar et al. investigated the impact of nicotine on feeding behaviour and associated hormonal changes [25]. Post hoc analysis indicated significant increases in food intake for nicotine, while GLP-1 coupled administration did not significantly alter food intake [25].

### Clinical data

Clinical data evaluating the efficacy of GLP-1RAs for NUD and nicotine use is preliminary (Table 4). The search identified three clinical studies with a total of 594 participants.

Yammine et al. evaluated craving and post-nicotine cessation body weight in smoking persons with prediabetes or obesity (n = 84) who were adjunctively assigned to placebo or extended-release exenatide in combination with NRT or no medication [26]. After six weeks of intervention, exenatide increased the incidence of smoking abstinence by 19.5%, demonstrating higher posterior probability compared to placebo (PP = 96.5%) [26]. The Questionnaire of Smoking Urges (QSU), which measures craving for nicotine revealed significantly lower urges to smoke in persons assigned to exenatide when compared to placebo [26]. Notably, there was a substantial difference in post-nicotine cessation body weight between the two groups, wherein the experimental group weighed 5.6 pounds less than placebo-treated participants at study endpoint [26].

A separate study enrolled 255 participants with moderate nicotine dependence and investigated whether adjunctive dulaglutide would aid in smoking cessation [27]. Participants were randomly assigned to either placebo or dulaglutide in combination with varenicline and behavioural counselling as part of a 12-week intervention period, with follow-up visits at weeks 24 and 52 [27]. The primary outcome, self-reported and biochemically confirmed abstinence at week 12, revealed no significant difference between the dulaglutide and placebo groups, with 63% and 65% abstinence rates, respectively [27]. Notwithstanding the non-significant difference between groups, participants assigned to dulaglutide exhibited lower post-nicotine cessation weight gain at the 12-week mark (− 1.0 kg) than the placebo group (+ 1.9 kg) [27].

The long-term effect of GLP-1RAs on nicotine abstinence, as well as rates of return-to-smoking behaviour, has also been preliminarily evaluated. Luthi et al. conducted a 12-month follow-up study that included participants who underwent a smoking cessation intervention program with dulaglutide or placebo in combination with standard care [28]. At week 52, prolonged abstinence rates were 29% and 27% for dulaglutide and placebo, respectively [28]. Collectively, findings suggest that GLP-1RAs may actively mitigate smoking cessation success, long-term abstinence, and post-nicotine cessation weight gain with overall good acceptability  [20–28].

## Discussion

Nicotine consumption as well as NUD continue to be prevalent and serious public health problems. The health impact of nicotine consumption is asymmetric in the general population insofar as higher rates of smoking are reported in minority groups, economically disadvantaged as well as persons living with mental disorders [29]. Some success has been achieved with public health campaigns that endeavour to reduce smoking behaviour in the general population. Notwithstanding, the impact of public health initiatives has been less pronounced in minority groups and persons with mental disorders wherein the rates of smoking remain higher (e.g., 40–80% smoking rates amongst persons with depressive, bipolar and psychotic disorders) [29]. The identification of a mechanistically informed acceptable and safe therapeutic capable of reducing craving, withdrawal, hyperphagia and post-cigarette smoking cessation weight gain would be potentially very impactful in subpopulations as well as the general population.

The search identified a total of eight preclinical (n = 5) and clinical (n = 3) studies evaluating the efficacy of GLP-1RAs for nicotine cessation. GLP-1RAs are FDA-approved drugs for obesity and T2DM and have been broadly studied as potential therapeutic agents in disorders characterized by alterations in reward processing pathways [10, 21–23, 27]. Results from preclinical studies indicate that endogenous and exogenous GLP-1 reduces dopamine in synapses and promotes the potentiation of GABAergic neurotransmitter release via MHb-IPN circuits, attenuating the reinforcing properties of nicotine [22]. Results from preclinical studies have reported that select GLP-1RAs meaningfully attenuate nicotine self-administration behaviours and reinstatement in rodents [20, 21].

The aforementioned findings are hypothesized to be subserved by modulation of the mesolimbic dopamine system and reductions in nicotine-induced dopamine release through the expression of dopamine reuptake receptors [11, 14]. In persons living with mental disorders , endogenous peripheral GLP-1 levels are reported to be lower than levels in serum in the general population, suggesting a potential mechanistic explanation for the higher rate of NUD as well as alcohol- and substance-use disorder in this population [28]. We propose that mental  GLP-1RAs modify  nicotine-induced effects on the mesolimbic dopamine system and consequently decreasing nicotine intake.

GLP-1RAs have been insufficiently evaluated as treatments for NUD. Results synthesized herein indicate that the adjunctive administration of GLP-1RAs may result in improved cessation rates compared to placebo. There is, however, inadequate characterization of the long-term effects of GLP-1RAs in persons with NUD. Available evidence also indicates that GLP-1RAs are able to mitigate post-cessation weight gain [20, 21, 23, 24, 26, 27]. Separately, it is reported that GLP-1RAs mitigate weight increase incurred from psychotropic drug exposure [24]. Additionally, GLP-1RAs show meaningful effects on nicotine consumption-related comorbidities in persons with NUD (e.g., cardiovascular disease) [30, 31].

There are several methodological limitations that affect inferences and interpretations of our analysis. The overarching limitation is that most studies are preclinical and mechanistic and the extent to which they translate to human populations and behaviours is uncertain. In addition, there are no large adequately designed randomized double-blind placebo-controlled studies that have evaluated GLP-1RAs as monotherapy in the treatment of NUD. Additionally, two of the identified clinical studies evaluate the same group of participants in a 12-month follow up study, limiting population diversity. Consequently, the evidence base regarding GLP-1RAs for nicotine use would be inadequate currently to inform decisions in the clinical setting wherein NUD is a therapeutic target. Nonetheless, potential benefits of GLP-1RAs on aspects of NUD may be observed in some persons taking these agents   for on-label indications (e.g., obesity, T2DM). Predisposition and/or prior history of addictive behaviours including NUD is an enduring trait inviting the need for longer-term studies. Also, only two GLP-1RAs (e.g., exenatide, dulaglutide) have been evaluated henceforth in NUD and the extent to which the efficacy of GLP-1RAs as a class effect is unknown. Moreover, the introduction of glucose insulinotropic polypeptide (GIP)/GLP co-agonists has yet to be studied in the treatment of NUD. Finally, there is a need for future studies to comprehensively ascertain clinical and/or biosignature aspects associated with health outcomes and tolerability in persons living with NUD. 

## Conclusion

GLP-1RAs show promise as potential treatments for persons with NUD, targeting smoking behaviour directly, craving, withdrawal, post-nicotine cessation weight gain, and tobacco-related comorbidities. Recent evidence indicates that GLP-1RAs are not causally related to suicidality, a critical safety observation in light of higher suicide rates amongst persons who are smokers living with mental illness [32]. Results from preclinical and preliminary clinical data justify conducting large, adequately controlled studies evaluating the short- and long-term outcome of GLP-1RAs in NUD. In addition, future studies should evaluate sub-populations with especially high rates of nicotine consumption (e.g., bipolar disorder), [33]. For practitioners providing care to persons with conditions wherein GLP-1RAs are indicated (e.g., obesity, T2DM) and who are smokers, awareness of the potential salutary effects of GLP-1RAs on smoking should be evaluated. In addition, future research vistas should attempt to ascertain whether GLP-1RAs not only reduce smoking behaviour but may also improve integrated outcomes involving mental health, quality of life and predisposition to use of other drugs of abuse.

## Data Availability

No datasets were generated or analysed during the current study.

## Abbreviations

GLP-1: Glucagon-like peptide-1
GLP-1RA: Glucagon-like peptide-1 receptor agonist
NUD: Nicotine use disorder
T2DM: Type 2 diabetes mellitus
NRT: Nicotine replacement therapy
NTS: Nucleus tractus solitarius
DAT: Dopamine reuptake transporter
MHb-IPN: Medial habenula-interpeduncular nucleus
